# The Serine Protease HTRA-1 Is a Biomarker for ROP and Mediates Retinal Neovascularization

**DOI:** 10.3389/fnmol.2020.605918

**Published:** 2020-11-17

**Authors:** Leah A. Owen, Kinsey Shirer, Samuel A. Collazo, Kathryn Szczotka, Shawna Baker, Blair Wood, Lara Carroll, Benjamin Haaland, Takeshi Iwata, Lakshmi D. Katikaneni, Margaret M. DeAngelis

**Affiliations:** ^1^Department of Ophthalmology and Visual Sciences, University of Utah, Salt Lake City, UT, United States; ^2^Department of Ophthalmology, Medical University of South Carolina, Charleston, SC, United States; ^3^School of Medicine, Medical Sciences Campus, University of Puerto Rico, San Juan, Puerto Rico; ^4^Department of Obstetrics and Gynecology, Huntsman Cancer Institute, University of Utah, Salt Lake City, UT, United States; ^5^Center for Clinical and Translational Science, University of Utah, Salt Lake City, UT, United States; ^6^Department of Population Health Sciences, University of Utah, Salt Lake City, UT, United States; ^7^National Institute of Sensory Organs, National Hospital Organization Tokyo Medical Center, Tokyo, Japan; ^8^Department of Pediatrics, Division of Neonatology, Medical University of South Carolina, Charleston, SC, United States; ^9^Department of Pharmacotherapy, The College of Pharmacy, University of Utah, Salt Lake City, UT, United States; ^10^Department of Ophthalmology, Jacobs School of Medicine and Biomedical Sciences State University of New York, Buffalo, NY, United States

**Keywords:** retinopathy of prematurity, biomarker, systems biology, preeclampsia, HTRA-1

## Abstract

Retinopathy of prematurity (ROP) is a blinding aberrancy of retinal vascular maturation in preterm infants. Despite delayed onset after preterm birth, representing a window for therapeutic intervention, we cannot prevent or cure ROP blindness. A natural form of ROP protection exists in the setting of early-onset maternal preeclampsia, though is not well characterized. As ischemia is a central feature in both ROP and preeclampsia, we hypothesized that angiogenesis mediators may underlie this protection. To test our hypothesis we analyzed peripheral blood expression of candidate proteins with suggested roles in preeclamptic and ROP pathophysiology and with a proposed angiogenesis function (HTRA-1, IGF-1, TGFβ-1, and VEGF-A). Analysis in a discovery cohort of 40 maternal-infant pairs found that elevated HTRA-1 (high-temperature requirement-A serine peptidase-1) was significantly associated with increased risk of ROP and the absence of preeclampsia, thus fitting a model of preeclampsia-mediated ROP protection. We validated these findings and further demonstrated a dose-response between systemic infant HTRA-1 expression and risk for ROP development in a larger and more diverse validation cohort consisting of preterm infants recruited from two institutions. Functional analysis in the oxygen-induced retinopathy (OIR) murine model of ROP supported our systemic human findings at the local tissue level, demonstrating that HtrA-1 expression is elevated in both the neurosensory retina and retinal pigment epithelium by RT-PCR in the ROP disease state. Finally, transgenic mice over-expressing HtrA-1 demonstrate greater ROP disease severity in this model. Thus, HTRA-1 may underlie ROP protection in preeclampsia and represent an avenue for disease *prevention*, which does not currently exist.

## Introduction

Retinopathy of prematurity (ROP) represents a significant risk of childhood blindness and demonstrates increasing incidence with improved survival of low birth-weight infants (Binenbaum et al., 2012; Quinn, 2016). There is no cure for ROP and our current treatments are associated with significant visual and ocular morbidity including severe myopia, retinal scarring, neurosensory retinal damage with loss of visual function, and cataract (O’Keefe et al., 1998; Cryotherapy for Retinopathy of Prematurity Cooperative Group, 2001; Good, 2004). Further, the treatment of ROP disease is unable to facilitate normal retinal vascularization and proper development and function of the neurosensory retina. ROP demonstrates a delayed onset of approximately 4–8 weeks following preterm birth, representing an opportunity for disease prevention rather than treatment. However, an incomplete understanding of systemic and neurosensory retinal molecular changes occurring before the manifestation of clinical disease prohibits this clinical approach. A greater understanding of ROP risk may better inform prevention strategies, however to date we and others have substantiated that only low birth weight (BW), early gestational age (GA), and post-natal oxygen exposure confer independent ROP risk (Slidsborg et al., 2016; Owen et al., 2017). While post-natal oxygen is the most modifiable risk, limiting post-natal oxygen may confer higher mortality (Carlo et al., 2010; Stenson et al., 2013; Owen and Hartnett, 2014). Beyond addressing these risk factors directly, numerous therapeutic interventions have been evaluated for efficacy in ROP prevention, though none have shown significant benefit as reviewed by Carroll and Owen (Carroll and Owen, 2020). As a result, current interventions are unable to modify risk and instead target ROP once the retinal disease is present and we are unable to restore normal retinal architecture and visual function. Therefore, we need a better understanding of *early* molecular changes mediating ROP disease to achieve meaningful progress toward the prevention of ROP blindness.

Herein, we take a novel approach to understanding early ROP molecular pathophysiology based on the clinical observation that early-onset maternal preeclampsia, characterized by placental insufficiency, is a natural model of ROP protection. While the molecular basis for observed ROP protection is not clear, this relationship has considerable support in the literature, including in a recent systematic review and meta-analysis (Fortes et al., 2011; Yu et al., 2012; Yau et al., 2015, 2016; Shulman et al., 2017; Azami et al., 2018; Morsing et al., 2018; Razak et al., 2018; Marins et al., 2019). Furthermore, this protective relationship is substantiated in animal models of preeclamptic and ROP disease (Fung et al., 2011; Becker et al., 2017). We hypothesize that a greater understanding of the molecular basis for ROP protection will facilitate therapeutically-mediated ROP prevention, resulting in decreased blinding effects of ROP disease. As ischemia is a central feature in both ROP and preeclampsia, we further hypothesized that angiogenesis mediators play a role in the observed ROP protection. To test this hypothesis we adopted a candidate approach, analyzing the systemic expression of proteins suggested to play a role in either preeclamptic or ROP pathophysiology and with a direct or indirect function in angiogenesis. These proteins included TGFβ-1, VEGF-A, IGF-1, and HTRA-1 (Muy-Rivera et al., 2004; Bergmann et al., 2010; Powers et al., 2010; Marzioni et al., 2012; Zhang et al., 2012; Dong et al., 2015; Hentges et al., 2015b; March et al., 2015; Teoh et al., 2015; Liao et al., 2017; Ferrari et al., 2009).

In the present study, we take a highly translational approach to test our hypothesis, evaluating the expression of each candidate protein in a discovery cohort of maternal/infant pairs and a validation cohort of preterm infants from two institutions. We further validate our findings through multiple lines of evidence in animal models to better understand the molecular mechanisms underlying preeclampsia-mediated ROP protection.

## Materials and Methods

### Participants

Study participants were enrolled in the Maternal Infant Pair Discovery Cohort or the ROP Replication Cohort as described below. All studies were performed with appropriate IRB approval at the University of Utah or the Medical University of South Carolina and adhered to the Declaration of Helsinki. All study participants (or legal guardians) were provided with informed consent before enrollment. Specific inclusion and exclusion criteria are as follows and consistent with AAP and American College of Obstetricians and Gynecologists guidelines (ACOG Committee on Practice Bulletins—Obstetrics, 2002).

#### Maternal Infant Pair Discovery Cohort

Maternal infant pairs with or without preeclampsia from one institution, the University of Utah, were recruited for enrollment at the time of preterm labor according to the following inclusion and exclusion criteria.

##### Mothers

Women with or without a diagnosis of preeclampsia as defined by pregnancy-induced hypertension >140/90 on two separate readings after 20 weeks gestation (ACOG Committee on Practice Bulletins—Obstetrics, 2002) were enrolled if they delivered infants before 31 weeks GA, therefore meeting ROP screening guidelines for our neonatal intensive care unit (NICU).

##### Infants

Infants born before 31 weeks and meeting GA and BW ROP screening criteria without known congenital ocular anomaly or delivery in the setting of chorioamnionitis (Owen et al., 2017).

#### ROP Validation Cohort

Infants were recruited from two NICUs: (1) the University of Utah or (2) the Medical University of South Carolina. Infants were included if they were born less than 31 weeks GA and meeting ROP screening guidelines at their respective centers and were eligible for blood collection.

### Study Protocol

Maternal infant pairs meeting inclusion and exclusion criteria were recruited in collaboration with the University of Utah Obstetrics and Gynecology Research Network and maternal peripheral blood collected within 24 h of preterm delivery. Infant cord blood was collected at the time of preterm delivery when deemed appropriate by the clinical care team. If cord blood was not collected for an enrolled infant, peripheral blood was collected in a piggy-back fashion within the first 7 days of life. Preterm infant peripheral blood was collected between 28 and 33 weeks post-menstrual age for Discovery Cohort infants and 30–35 weeks for ROP Cohort infants. Per our standardized protocol, up to 2 ml of infant blood was collected and plasma immediately separated, accessioned, and stored at −80°C. Epidemiologic, demographic, and clinical data including preeclampsia, maternal age, preterm labor, race/ethnicity, need for infant surgery <30 weeks, infant sex, infant GA, and BW were collected for each enrolled patient and stored in a secure REDCap database.

### Outcome Measures for Human Studies

The primary outcome variables were the presence or absence of any ROP or maternal preeclampsia as previously defined (Good and Hardy, 2001; ACOG Committee on Practice Bulletins—Obstetrics, 2002). The diagnosis of preeclampsia was determined from the medical chart and verified using gold-standard diagnostic criteria from the American College of Obstetricians and Gynecologists (ACOG Committee on Practice Bulletins—Obstetrics, 2002). ROP zone, stage, and severity (including assessment of plus and pre-plus disease) were documented according to the international classification of ROP and determined by indirect ophthalmoscopy provided in the course of clinical care (Good and Hardy, 2001; International Committee for the Classification of Retinopathy of Prematurity, 2005).

### Univariate Candidate Protein Analysis

Expression of each candidate protein was measured in maternal or infant plasma using either ELISA [HTRA-1 (LSBio #LS-F11673), IGF-1 (R&D #DG-100)] or Luminex (TGFβ-1 and VEGF-A) when a clinical test was available through the ARUP clinical laboratory (ARUP Laboratories, 2020 | ARUP Laboratories, n.d.). All ELISA samples were run in triplicate. Average values for each condition were compared for statistical significance using a traditional two-tailed *t*-test.

### Multivariate HTRA-1 Statistical Analysis

The association between each candidate factor and ROP status was assessed *via* logistic generalized estimating equations (GEE) models, accounting for patient-wise clustering *via* robust sandwich estimation of variance. The ability of potential predictive factors to discriminate between patients with and without ROP was assessed in terms of area under the receiver operating characteristic curve (AUC), with confidence intervals and *p*-values computed *via* 10,000 patient-wise bootstrap samples.

### Animal Studies

Under approved IACUC protocol 16-09005, wild-type C57B6, or HtrA-1 over-expression transgenic mice (HtrA-1Tg; Nakayama et al., 2014) were placed in the oxygen-induced retinopathy (OIR) model of ROP (Stahl et al., 2010; Iejima et al., 2015b). A minimum of four biologic replicates, representing eight eyes, were performed for each condition: HtrA-1Tg RA; HtrA-1Tg OIR; C57B6 RA; C57B6 OIR. Two technical replicates were performed for each condition. Consistent with the OIR protocol as published (Connor et al., 2009), at p17 the mice were humanely euthanized and immediately following globes enucleated and fixed in 4% PFA in PBS. Retinas were isolated from fixed globes using a dissection microscope. The vasculature was then stained using Isolectin-594 and imaged in flat-mount preparation. Retinal images were obtained using an Invitrogen EVOS M7000 Imaging System. Quantification of neovascularization was done as described (Connor et al., 2009). In brief, we performed masked tracings of the neovascular retina, avascular retina, and total retinal area such that areas of neovascularization were quantified relative to the total retinal area for comparison between conditions (Connor et al., 2009).

### Murine Retinal and Retinal Pigment Epithelium HtrA1 RT-PCR

Wild-type C57BL6 pups were placed under OIR or room air (RA) conditions and retinal and RPE tissues dissected using a dissecting microscope as described above (Owen et al., 2019). Tissues from one eye per animal were used and three biologic replicates were pooled; a minimum of three technical replicates was performed for each condition. RT-PCR was performed using commercially available Taqman Gene Expression Assay m1 for mouse HTRA-1 (#Mm00479892). Fold change was determined after normalization to GAPDH.

## Results

### Maternal HTRA-1 and IGF-1 Expression Are Significantly Decreased in Early-Onset Preeclampsia

To determine if angiogenesis mediators underlie the basis of ROP protection in maternal preeclampsia we identified candidate proteins for analysis, which have described roles in preeclampsia and retinal neovascular disease as well as indirect or direct roles in angiogenesis. These proteins included TGFβ1 (Muy-Rivera et al., 2004; Ferrari et al., 2009; Yingchuan et al., 2010), VEGF-A (Dong et al., 2015; Hentges et al., 2015a; Gaynon et al., 2018), IGF-1 (Bańkowski et al., 2000; Hellstrom et al., 2001; Hellström et al., 2016; Liao et al., 2017) and HTRA-1 (Deangelis et al., 2008; Zhang et al., 2012; Hasan et al., 2015; Teoh et al., 2015). We prospectively recruited and followed a discovery cohort consisting of 40 maternal/infant pairs. The maternal analysis consisted of women delivering before 31 weeks gestation in the presence (*n* = 15) or absence (*n* = 25) of early-onset maternal preeclampsia. Clinical features, as depicted in Table 1, were similar between groups except for a higher proportion of Hispanic women and preterm labor in participants without preeclampsia. We assessed systemic levels of each candidate protein in plasma at the time of preterm birth using ELISA (HTRA-1 and IGF-1) or Luminex (TGFβ-1 and VEGF-A) analysis platforms. As demonstrated in Table 2, we found no significant difference in average TGFβ-1 or VEGF-A expression relative to preeclampsia. IGF-1 and HTRA-1 demonstrated significantly lower levels of systemic expression in women with early-onset preeclampsia vs. women without preeclampsia. The presence or absence of preterm labor was not significantly associated with the expression of either IGF-1 or HTRA-1.

**Table 1:** Discovery cohort clinical characteristics.

	Early onset preeclampsia (*n* = 15)	No preeclampsia (*n* = 25)		
Maternal characteristics	% Cohort/Average	% Cohort/Average		
Presence of Preterm Labor*	13	72		
Antenatal steroid administration	67	60		
Maternal Age	34.7 (R: 27–43)	32.7 (R: 21–40)		
*Race*
Caucasian	67%	64%		
Black or African American	0%	4%		
Native Hawaiian or Pacific Islander	0%	4%		
Undisclosed	13%	12%		
*Ethnicity*
Hispanic	13%	44%		
Non-hispanic	87%	56%	
	**No ROP (13)**	**Any ROP (*n* = 18)**	**Maternal Preeclampsia (*n* = 12)**	**No Preeclampsia (*n* = 19)**
Infant characteristics	% Cohort/Average	% Cohort/Average	% Cohort/Average	% Cohort/Average
Need for surgery <30 weeks	8	6	0	10.5
Birth weight (grams)	1,161.6 g (R: 645–1,650 g)	831.6 g (R: 460–1,360 g)	797.9 g (R: 460–1,300 g)	1,074.2 g (R: 650–1,650)
Gestational age (weeks)	29.14 (R: 26.71–31.0)	26.43 (R: 24.0–28.71)	27.57 (R: 25.14–30.43)	27.57 (R: 24.0–31.0)
Male sex	38	44	33	47

*Clinical and demographic characteristics of 40 maternal/infant pairs consisting of women delivering before 31 weeks gestation in the presence or absence of early-onset maternal preeclampsia.

**Table 2:** Average systemic values of candidate angiogenic factors by ELISA or Luminex*.

Angiogenic factor	Average value in peripheral circulation (pg/ml)					
	Maternal preeclampsia	Maternal control	*p*-value	Preterm labor	Absence of labor	*p*-value
IGF-1	125,673	219,624	0.014	202,813	142,484	0.101
TGFβ-1*	1,323.11	1,181.84	0.372			
VEGF-A*	21.11	22.07	0.454			
HTRA-1	1,956,789	8,128,232	0.052	5,123,743	5,605,944	0.487

Candidate proteins were measured in the peripheral circulation of women within the discovery cohort delivering between 24 and 31 weeks gestation. Peripheral blood samples were collected within 24 h of delivery. Average values for each candidate protein were stratified by the presence (*n* = 15) or absence (*n* = 25) of maternal preeclampsia. Only systemic IGF-1 (*p* = 0.014) and HTRA-1 (*p* = 0.052) were found to be significantly different in women with as compared to without preeclampsia. Values demonstrating significance were further stratified by the presence or absence of preterm labor. Neither IGF-1 (*p* = 0.101) or HTRA-1 (*p* = 0.487) expression was found to differ significantly with the presence or absence of preterm labor. *Proteins followed by an asterisk in the table are Luminex based versus the others which are ELISA based.

### Preterm Infant HTRA-1 Expression Is Significantly Associated With Maternal Preeclampsia and the Development of ROP

We next assessed preterm infant expression of each candidate protein relative to ROP and maternal preeclampsia within our discovery cohort. We identified 18 infants with and 13 infants without ROP; 12 infants were born in the presence of and 19 infants born in the absence of maternal preeclampsia. Clinical characteristics as represented in Table 1, were overall similar between groups. We found a statistically significant difference in average infant systemic HTRA-1, but not TGFβ-1, IGF-1, or VEGF-A relative to the presence or absence of ROP and maternal preeclampsia. Specifically, we found that HTRA-1 protein expression was significantly decreased in the systemic circulation of infants born in the setting of preeclampsia when measured in a standardized GA window of 28–33 weeks (Table 3). Further, systemic infant HTRA-1 expression was significantly increased in infants with ROP relative to those without within this same GA window (Table 3). To determine if this association was influenced by post-natal age, we analyzed the umbilical cord, the first week of life, and 28–33 week peripheral blood samples from infants within the discovery cohort. We found that the association between elevated HTRA-1 and subsequent development of ROP was most significant during the first 5 weeks after birth *p* = 0.065. To determine if we could replicate these findings, we analyzed the significance of systemic HTRA-1 expression for ROP development in a second, validation cohort consisting of 100 infants recruited from two institutions. Clinical characteristics are represented in Table 4. Systemic HTRA-1 expression was measured using ELISA in infants with (*n* = 42) or without (*n* = 58) ROP from plasma collected during GA weeks 30–35. As demonstrated in Table 3, HTRA-1 expression remains significantly elevated in infants with ROP in this larger cohort.

**Table 3:** Average preterm infant systemic values of candidate angiogenic factors by ELISA or Luminex*.

Angiogenic factor	Average value in peripheral circulation at GA weeks 28.43–33.0 weeks (pg/ml)						Average value in peripheral circulation (30.57–34.86 weeks)		
	Maternal preeclampsia	Maternal control	*p*-value	Any infant ROP	No infant ROP	*p*-value	Any infant ROP	No infant ROP	*p*-value
IGF-1	19,511.00	25,120.00	0.163	24,021.00	20,477.00	0.292			
TGFβ-1*	3,359.67	3,983.20	0.349	4,011.43	4,882.71	0.285			
VEGF-A*	116.30	237.30	0.138	218.60	203.1	0.452		
HTRA-1	153,717.60	274,647.79	0.001	338,066.23	188,185.27	0.01	353,371.98	216,836.76	0.002

Candidate proteins were measured between 28.43–33 weeks GA in the peripheral circulation, of preterm infants delivering between 24 and 31 weeks gestation in the Discovery Cohort. Average values were stratified by the presence (*n* = 12) or absence (*n* = 19) of maternal preeclampsia and the development (*n* = 18) or absence (*n* = 13) of infant ROP. Only systemic HTRA-1 expression was found to be significantly different relative to maternal preeclampsia (*p* = 0.001) and preterm infant retinopathy of prematurity (ROP; *p* = 0.01). HTRA-1 expression was subsequently measured between 30–35 weeks in the Replication Cohort; analysis was stratified by the presence (*n* = 42) or absence (*n* = 58) of infant ROP. This analysis replicated findings in the Discovery cohort showing that HTRA-1 expression is significantly elevated (*p* = 0.02) in infants with ROP as compared to those without. *Proteins followed by an asterisk in the table are Luminex based versus the others which are ELISA based.

**Table 4:** Replication cohort clinical characteristics.

	Replication cohort	
	No ROP (*n* = 58)	Any ROP (*n* = 42)
Infant characteristics	% Cohort/Average	% Cohort/Average
Birth weight	1,150.5g (R: 735–1,465g)	759.4 g (R: 445–1,080 g)
Gestational age	26.86 (R: 25.43–31.29)	26.00 (R: 23.14–32.43)
Male sex	19%	26%
* Race*
Caucasian	62%	29%
Black or African American	48%	52%
Native Hawaiian or Pacific Islander	0%	0%
Undisclosed	3%	4%
*Ethnicity*
Hispanic	18%	12%
Non-hispanic	82%	88%

*Clinical characteristics of 100 preterm infant delivering before 31 weeks gestation from two institutions with (*n* = 42) or without (*n* = 58) subsequent development of ROP*.

### Preterm Infant HTRA-1 Expression Is Significantly Associated With Subsequent ROP Development

**Figure 1:**
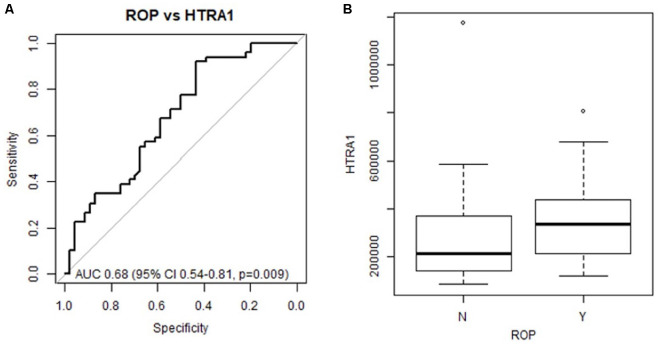
Preterm infant HTRA-1 predicts subsequent retinopathy of prematurity (ROP) development in a dose-dependent fashion. HTRA-1 protein was measured using ELISA in preterm infant peripheral (*n* = 140) and cord blood (*n* = 36) samples. **(A)** The area under the receiver operating curve (AUC) demonstrates the significance of average HTRA-1 values for the prediction of ROP development (*p* = 0.009). **(B)** Odds ratio analysis demonstrates an increase of 2.32 ROP risk for each doubling of systemic HTRA-1 expression (95% CI 1.03–5.24, *p* = 0.043).

To determine if systemic HTRA-1 expression is independently associated with ROP development and can predict subsequent preterm infant ROP development we performed a multivariate analysis of peripheral blood samples from the described cohorts. Analysis of area under the receiver operating characteristic curve (AUC), determined that systemic HTRA-1 expression was significantly predictive of subsequent ROP development (*p* = 0.009) for all GA groups when controlling for the significance of known risk factors including BW and GA (Figure 1A). Additionally, we found that systemic HTRA-1 expression increased infant ROP risk in a dose-dependent fashion. As demonstrated in Figure 1B, we found that the likelihood of ROP increases by an odds ratio of 2.32 (95% CI 1.03–5.24, *p* = 0.043) with each doubling of systemic HTRA-1. In all analyses, we found no interaction between GA or BW with HTRA-1 expression.

### HtrA-1 Expression Is Increased in the Murine Retina and Retinal Pigment Epithelium in the Oxygen-Induced Retinopathy Model of ROP

To determine if HTRA-1 plays a functional role in retinal neovascular pathology in ROP we evaluated neurosensory retinal and retinal pigment epithelial (RPE) HtrA-1 expression in the OIR murine model of ROP (Connor et al., 2009). Wild-type C57B6 p7 pups were placed under experimental OIR or control RA conditions and neurosensory retinal or RPE HtrA-1 expression quantified using RT-PCR. We found, as shown in Table 5, that mice under OIR conditions developed the expected ROP phenotype and exhibited increased expression of HtrA-1 in both the retinal and RPE tissues as demonstrated by an increased fold change.

**Table 5:** Murine retinal and retinal pigment epithelial (RPE) tissues express greater levels of HtrA-1 under oxygen-induced retinopathy (OIR) compared with control conditions.

Treatment condition	HtrA-1 expression fold change under OIR vs. room air conditions
Retinal pigment epithelium	1.67
Neurosensory retina	1.17

*Murine retinal and RPE tissues were dissected following the induction of ROP using the OIR model or control conditions. RT-PCR was performed on four biologic replicates from four animals per condition. Results were normalized to GAPDH and reported as fold change between treatment and control conditions*.

### Transgenic Mice Over-Expressing HtrA-1 Have a More Severe Retinal Neovascular Disease in the OIR Model

**Figure 2:**
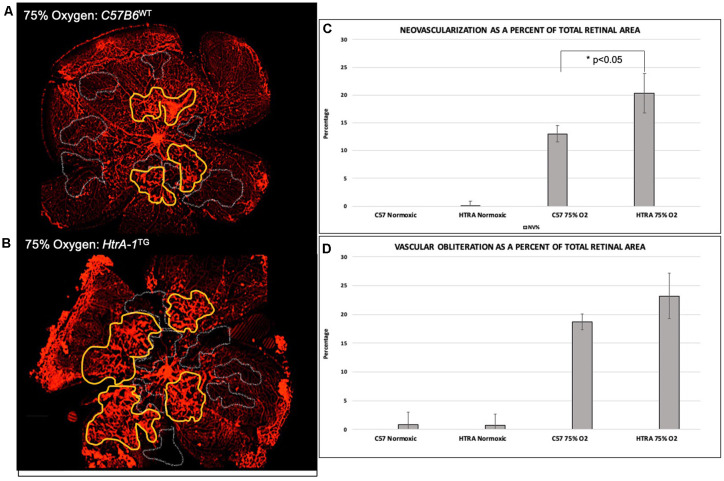
Transgenic mice over-expressing HtrA-1 demonstrate greater severity of neovascularization in the oxygen-induced retinopathy (OIR) model. HtrA-1Tg or Wild-type C57B6 mice were placed under OIR or RA conditions (*n* = 8 mice per genetic background per condition). **(A,B)** Neovascularization (NV) and vascular obliteration (VO) were assessed using Isolectin-594 staining of flat-mount images. Representative areas of NV and VO have been highlighted in yellow and gray respectively. **(C,D)** Quantification of NV **(C)** and VO **(D)** was done using masked tracings of the neovascular retina, avascular retina, and total retinal area such that NV and VO were quantified relative to the total retinal area.

As elevated HTRA-1 levels are associated with increased ROP in preterm infants, we sought to determine if elevated HtrA-1 mediates the retinal neovascular phenotype in ROP. To do so, we evaluated the retinal neovascular (NV) and vascular obliteration (VO) phenotypes of transgenic mice over-expressing HtrA-1 (HtrA-1Tg; Nakayama et al., 2014) in the OIR model of ROP. We found increased pre-retinal neovascularization in the HtrA-1Tg retina compared with the wild-type C57B6 control retina under OIR conditions (Figures 2A,B). No significant aberrancy of retinal vasculature was noted in either the wild-type C57B6 or HtrA-1Tg mice under RA conditions. When analyzed relative to the total retinal area, we found a statistically significant difference in the neovascular area of the HtrA-1Tg vs. wild-type mouse (*p* < 0.05; Figure 2C). Though there was a trend toward significance, we did not find a statistically significant difference in the area of vascular obliteration between conditions (Figure 2D).

## Discussion

ROP accounts for significant childhood blindness in the US and worldwide (Gilbert and Muhit, 2008). The scope of the problem is increasing with the improved viability of early-term births, particularly in developing nations (Quinn et al., 2010; Quinn, 2016). Our clinical care for this vulnerable population has two significant knowledge gaps. First, our screening methods lack specificity; currently, only approximately 50% of infants screened with developing ROP (Binenbaum et al., 2012; Quinn et al., 2014; Daniel et al., 2015; Owen et al., 2017). Second, we are unable to prevent ROP disease and the gold standard treatment remains the destruction of the avascular neurosensory retina. Both ROP disease and treatment can have significant, life-long, visual sequela (Cryotherapy for Retinopathy of Prematurity Cooperative Group, 2001; Good, 2004). Therefore, we need to identify early biomarkers that improve the specificity of our screening tools and interventions that allow for disease prevention rather than mitigation once the disease is present.

To address these critical knowledge gaps, we studied a natural form of ROP protection found in the setting of early-onset maternal preeclampsia, investigating both human and animal models. As both disease states are typified by aberrant angiogenesis, we hypothesized that shared aberrancy in angiogenesis mediators may underlie the observed clinical paradigm of protection. TGFβ-1, VEGF, IGF-1, and HTRA-1 are unique in that they have all been postulated to play a role in ROP and preeclampsia and have a direct and indirect role in angiogenesis (Ajayi et al., 2008; Ferrari et al., 2009; Bergmann et al., 2010; Powers et al., 2010; Marzioni et al., 2012; Chen et al., 2014; Dong et al., 2015; Hentges et al., 2015a; Teoh et al., 2015; Liao et al., 2017; Liu et al., 2018). Herein we show that HTRA-1, but not TGFβ-1, VEGF, or IGF-1, may mediate ROP protection in the setting of early-onset preeclampsia. More specifically, we demonstrate that elevated systemic HTRA-1 expression is significantly associated with increased risk of preterm infant ROP and conversely that decreased systemic HTRA-1 expression is significantly associated with preeclampsia within a discovery cohort of maternal/infant pairs. This suggests that maternal preeclampsia may confer lower HTRA-1 expression, which is then protective for infant ROP development. Furthermore, when considering a second, ethnically diverse validation cohort, we confirm the significance of elevated systemic HTRA-1 expression to the development of ROP and show that the likelihood of ROP development relative to systemic HTRA-1 expression demonstrates a dose-response, specifically a 2.32 increased rate of ROP development for each doubling of systemic HTRA-1 expression. Importantly, the significance of HTRA-1 expression to ROP development remained after controlling for GA and BW, suggesting that HTRA-1 has potential value as an independent predictor of ROP. Interestingly, we also show that the difference in HTRA-1 expression between infants who subsequently develop ROP and those who do not is most significant in the first 5 weeks of life. This is compelling evidence that HTRA-1 expression may be influenced by preeclampsia and the *in utero* environment. Certainly, if decreased HTRA-1 is a function of preeclampsia, and somehow conferred to the infant resulting in ROP protection, we would expect this difference to be present at birth. Future studies are needed to fully assess this relationship and understand the mechanisms of HTRA1 regulation both pre and post-natally.

In addition to our identification of HTRA-1 as a biomarker for ROP disease, we also sought to understand if HTRA-1 expression is relevant to local ocular disease. Our functional analysis in wild-type and transgenic murine models substantiates our human data and further suggests a functional role for HtrA-1 in local disease. Specifically, we offer two lines of evidence that HtrA-1 participates in neurosensory retinal and RPE disease changes. First, we show that wild-type mice induced to develop ROP in the OIR model display elevated neurosensory retinal and RPE HtrA-1 expression by RT-PCR. Second, we specifically interrogate the hypothesis generated in our human studies that elevated HTRA-1 is correlated with ROP disease using the HtrA-1Tg murine model. In further support of a role for elevated ocular HtrA-1 in ROP pathomechanisms, we show that HtrA-1Tg mice demonstrate increased severity of pre-retinal neovascularization in the OIR model of ROP. Importantly, these findings are supported by the current understanding of HTRA-1 expression and ROP disease mechanisms. While there is a paucity of data regarding physiologic ocular HTRA-1 expression, published work suggests a predominance of expression in the RPE, consistent with our finding that the increase in HtrA-1 expression under OIR conditions was most apparent in the RPE (Chan et al., 2007). Further, HTRA-1 is a secreted protein and there is precedent for the RPE secretome in ROP and other retinal neovascular disease mechanisms as reviewed by Araújo et al. (2018). Therefore, the correlation between elevated systemic HTRA-1 and ROP in preterm infants may confer relevance to local disease mechanisms. Future work will clarify the specific disease importance of systemic vs. local HTRA-1. Certainly, systemic elevation of HTRA-1 could be a biomarker, acting as a surrogate for the local ocular environment. In this way measurement in preterm infants could allow for better disease prediction on the basis of molecular ocular state. However, it could also be that elevation of systemic HTRA-1 is necessary for the observed ocular phenotype and more directly affects the molecular ocular pathomechanisms.

Our work describing potential roles for HTRA-1 as a disease biomarker and mediator of ROP protection in preeclampsia is broadly supported in the literature. Certainly, there is precedent for systemic dysregulation of HTRA-1 in preeclampsia though the precise changes relative to gestation as well as maternal and infant disease are not well elucidated owing to the paucity of data and variable GA assessments (Ajayi et al., 2008; Marzioni et al., 2012; Chen et al., 2014; Teoh et al., 2015; Liu et al., 2018). To the best of our knowledge, our study is the first to analyze maternal HTRA-1 expression within an early-onset preeclampsia cohort delivering before 31 weeks and will lay the foundation for further such studies. Importantly, animal studies support a role for aberrancy of HtrA-1 expression in preeclampsia demonstrating that murine lack of HtrA-1 expression phenocopies placental insufficiency found in maternal preeclampsia (Hasan et al., 2015). Furthermore, HTRA1 genetic variation has been extensively associated with neovascular retinal disease in the setting of AMD (Dewan et al., 2006; Yang et al., 2006; Deangelis et al., 2008; Andreoli et al., 2009; Jacobo et al., 2013; Iejima et al., 2015b), with a preponderance of evidence suggesting that the disease is mediated by increased HTRA-1 expression (Jones et al., 2011; Iejima et al., 2015a,b; Tom et al., 2020). Finally, work in various areas supports a role for HTRA1 in angiogenesis. Several groups have described a role for HTRA-1 in vascular development as well as specifically in retinal angiogenesis (Zhang et al., 2012; Jacobo et al., 2013; Chen et al., 2018). The precise mechanism(s) by which HTRA-1 participates in angiogenesis is not fully elucidated, though work has demonstrated this may be through the canonical Wnt signaling pathway or in coordination with thrombospondin or the TGFβ family member GDF6 (Zhang et al., 2012; Chen et al., 2018; Klose et al., 2018). This is evidenced on a functional level as systemic changes in HtrA-1 in murine tumor models lead to aberrancy of tumor vascularization (Klose et al., 2018). With greater relevance to retinal neovascularization, recent work has demonstrated at the *in vitro* and *in vivo* level that HTRA-1 signaling *via* the Wnt pathway mediates VEGF expression in RPE cells and further, that HTRA-1 inhibition results in decreased choroidal neovascularization in murine disease models (Lu et al., 2019). Taken together, these data suggest it is plausible that HTRA-1 can facilitate both preeclampsia-mediated ROP protection and participate in local neovascular ROP pathophysiology.

In summary, our work is complementary and builds on the current foundation of knowledge, using a highly translational human analysis of a natural form of ROP protection with targeted functional analysis in animal models. This is a unique paradigm of clinical protection with an unclear molecular basis. If known, we could change ROP management from one of destructive treatment to protection. Our work identifies a novel role for the serine protease HTRA-1 as a potential mediator of ROP protection in the setting of early-onset preeclampsia and a dose-dependent biomarker for ROP. Further, we use multiple lines of evidence in murine models to demonstrate a functional role for HtrA-1 in local disease mechanisms. Thus, our data suggest a role for HTRA-1 in preeclampsia-mediated ROP protection which can be assessed in the systemic circulation. While further studies are needed to fully characterize the molecular mediators of ROP protection in this setting, our work is an important step that may allow for increased specificity of ROP screening and early intervention with disease prevention. Future work will be directed at understanding mechanisms of HTRA-1 regulation both within the context of preeclampsia and in the neurosensory retina and developing retinal vasculature, allowing for targeted interventions.

## Data Availability Statement

Data generated in this study will be made available upon request by the authors in accordance with NIH data sharing policy and with appropriate IRB approvals.

## Ethics Statement

The studies involving human participants were reviewed and approved by the University of Utah and MUSC IRB boards. Written informed consent to participate in this study was provided by the participants’ legal guardian/next of kin. The animal study was reviewed and approved by University of Utah School of Medicine IACUC.

## Author Contributions

LO, MD, and LK: conceptualization. LO, BH, MD, SB, and KS: methodology. LO, MD, KS, LK, and TI: validation. LO, LC, SC, BW, KS, and MD: formal analysis. MD, LO, LK, and TI: resources. MD and LO: investigation, data curation, writing—original draft preparation, supervision, project administration and funding acquisition. All authors contributed to the article and approved the submitted version.

## Conflict of Interest

The authors declare that the research was conducted in the absence of any commercial or financial relationships that could be construed as a potential conflict of interest.
